# Suicide Risk Screening in a Diverse Cohort of Youth With Type 1 and Type 2 Diabetes

**DOI:** 10.1155/pedi/6662248

**Published:** 2025-06-02

**Authors:** Saleel Fatima, Laura Prichett, Nancy Campbell, Meg C. N. Snyder, Morgan Bifano, Risa M. Wolf

**Affiliations:** ^1^Division of Pediatric Endocrinology, Department of Pediatrics, Johns Hopkins University School of Medicine, Baltimore, Maryland, USA; ^2^Biostatistics, Epidemiology and Data Management (BEAD) Core, Johns Hopkins School of Medicine, Baltimore, Maryland, USA; ^3^Department of Pediatric Social Work, Johns Hopkins Hospital, Baltimore, Maryland, USA; ^4^Department of Behavioral Psychology, Kennedy Krieger Institute, Baltimore, Maryland, USA

**Keywords:** diabetes, suicide, youth

## Abstract

**Introduction:** Depression and suicide are more prevalent in adolescents with chronic illnesses such as diabetes. Psychosocial assessment is recommended in routine diabetes care. The goal of this study was to determine the prevalence of suicide risk in youth with diabetes and to determine the utility of the Patient Health Questionnaire-9 (PHQ-9) Item 9 compared to the Ask Suicide-Screening Questions (ASQ).

**Methods:** The PHQ-9 and ASQ were prospectively administered to patients with type 1 diabetes (T1D) and type 2 diabetes (T2D), ages 11–24 years at routine diabetes visits at a pediatric diabetes center from January to December 2023. Depression and suicide risk were assessed using PHQ-9 Item 9 and ASQ. The sensitivity and specificity of PHQ-9 were determined using ASQ as the reference standard.

**Results:** Among the 309 patients included in this study, 237 (76.6%) had T1D and 72 (23.3%) had T2D. The mean age was 15.1 ± 2.6 years, 145 (46.9%) were female, and the mean HbA1c was 8.6% ± 2.3%. The prevalence of suicide risk using PHQ-9 Item 9 was 5.9% in T1D and 12.5% in T2D, and 8.4% in T1D and 19.4% in T2D, using ASQ. The sensitivity of the PHQ-9 Item 9 was 55.9% (95% CI: 37.9, 72.8%), specificity was 98.5% (95% CI: 96.3, 99.6%), PPV was 82.6% (95% CI: 61.2, 95%), and NPV was 94.8% (95% CI: 91.5, 97%) as compared to ASQ as reference standard. After a positive suicide risk screen, only 52.9% completed mental health follow-up at 1 month. The feasibility survey showed providers could identify high risk patients without workflow impact.

**Conclusion:** Prevalence of suicide risk is higher in youth with T2D compared to T1D. PHQ-9 is less sensitive in identifying suicide risk in adolescents and young adults (AYA) compared to the ASQ. Diabetes care teams should consider using a specific suicide risk screener in routine diabetes care. Follow-up with mental health is suboptimal, and should be encouraged.

## 1. Introduction

Depression and suicidal ideation (SI) are prevalent in adolescents with chronic illnesses such as diabetes and may have negative effects on medication adherence and health-related outcomes [1]. The American Diabetes Association (ADA) and International Society of Pediatric and Adolescent Diabetes (ISPAD) recommend that children and adolescents with diabetes undergo routine assessment of quality of life, adjustment problems, depression, anxiety, disordered eating, diabetes distress, and other psychosocial issues of concern at planned intervals by mental health professionals as part of diabetes care [2, 3]. However, no specific guidelines exist for assessing suicide risk for adolescents and young adults (AYA) with diabetes, and few clinics incorporate mental health screening into their care model [4]. Higher depression rates have been identified in youth with diabetes when compared to their nondiabetic peers, and there are noted differences in depressive symptoms when comparing individuals with type 1 diabetes (T1D) and type 2 diabetes (T2D). In the pediatric T1D population, prevalence rates of mild and moderate depression have been reported as 6%–17%, and 5%–14%, respectively [5]. The Pediatric Diabetes Consortium (PDC), analyzing a group of eight academic centers in the United States, found that adolescents with T2D self-report a 50% higher rate of depressive symptoms compared to adolescents with T1D [6]. According to a recent study published in 2022, it is common for patients with youth-onset T2D to have diabetes distress, depressive symptoms, and anxiety symptoms [7]. Youth-onset T2D is linked to complications and psychosocial risks, disproportionately affecting marginalized and socioeconomically disadvantaged youth. Screening for depression in youth with diabetes largely focuses on T1D, with limited data on T2D [8]. Suicide risk prevalence ranges from 2.2% to 15.0% in AYA with T1D [9, 10], and 9%−18.9% in AYA with T2D [11–13]. There is more limited data available on youth with T2D, despite their higher depressive symptom rates compared to T1D. This gap leaves many at-risk youth unidentified and without access to mental health support [14].

There exists a variety of mental health screening tools to assess depression and suicide risk for AYA. The most commonly used depression screener in medical settings is the Patient Health Questionnaire-9 (PHQ-9) [15]. The PHQ-9 has eight items measuring depressive symptoms and one item (Item 9) assessing suicide risk [16]. Other screening tools include the Children's Depression Inventory (CDI) [17], Ask Suicide-Screening Questions (ASQ) [18], Columbia-Suicide Severity Rating Scale (C-SSRS) [19], and Suicidal Ideation Questionnaire/Suicidal Ideation Questionnaire-Junior (SIQ/SIQ-JR) [20, 21]. Among these, the ASQ stands out for its brevity, comprising only four yes/no questions, and has been validated in the youth population [22]. However, suicide risk screening using a suicide specific screener is not commonly integrated into routine diabetes clinic visits [23]. Thus, there is limited data on the prevalence of suicide risk in AYA with diabetes using a suicide specific screener, particularly in the youth onset T2D population. Addressing these gaps is essential to ensure comprehensive care and support for youth navigating diabetes management.

The objectives of this study were to determine the prevalence of suicide risk in AYA with diabetes using PHQ-9 Item 9 and ASQ, and to determine the diagnostic accuracy of the PHQ-9 suicide-risk item for identifying the presence of suicide risk in AYA with diabetes compared to the validated suicide-specific measure, ASQ.

## 2. Methods

### 2.1. Study Design and Eligibility

This was a prospective quality improvement study to improve suicide risk screening in the pediatric diabetes population, involving administration of the PHQ-9 and ASQ survey at routine diabetes clinic visits at a tertiary care pediatrics diabetes center (Johns Hopkins Children's Center) with an ethnically diverse population, from January to December 2023.

Patients with a diagnosis of T1D and T2D, and ages 11–24 years were included. Patients with a diagnosis of prediabetes, CFRD, steroid induced diabetes, as well as other significant comorbidities (e.g., heart failure, malignancy, mitochondrial disorder, and nephrotic syndrome), were excluded. Approval from the Johns Hopkins University Institutional Review Board was obtained, with a waiver of consent.

### 2.2. Study Procedures

At routine diabetes clinic visits, patients meeting inclusion criteria were given the PHQ-9 and ASQ questionnaires. PHQ-9 is administered as standard of care depression screening per ADA guidelines once per year. PHQ-9 is a depression screening tool with nine questions, with the final question (Item 9) being “thoughts that you would be better off dead or hurting yourself in some way,” which is a measurement of suicide risk. PHQ-9 assesses symptoms in the past 2 weeks. PHQ-9 scoring ranges from 5 to 9 for mild, 10–14 for moderate, 15–19 for moderately severe, and 20–27 for severe depression. The ASQ questionnaire consisting of four brief suicide screening questions (assessing risk in the past few weeks) was administered along with the routinely administered PHQ-9. Patients were asked to fill out these two questionnaires on paper in the clinic during their routine visit, and then results were entered and stored in the patient electronic medical record (EMR). An additional question was asked about family history of mental health disorders.

**Figure 1:**
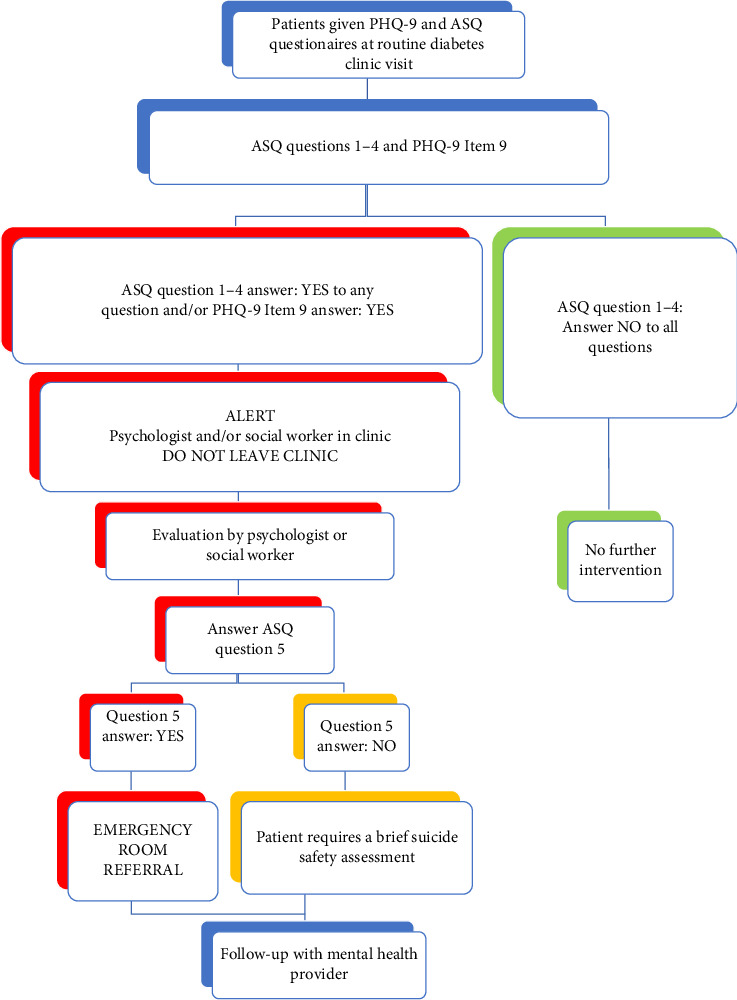
Workflow for depression and suicide screening in clinic.

The existing workflow (Figure 1) for depression screening was that if someone responded in the affirmative to item 9 on PHQ-9, they were assessed by a behavioral health psychologist or social worker for suicide risk during that visit. “Positive” screen on the PHQ-9 is considered answer 1, 2, or 3 on Item 9 on the PHQ-9. The ASQ suicide risk screener consists of four brief questions assessing recent suicidal thoughts (“wished you were dead”, “thoughts about killing yourself”), feelings of worthlessness (“felt that you or your family would be better off if you were dead”), and Question 4 asks about past suicide attempts (“ever tried to kill yourself”). Similarly, with implementation of ASQ screening, if someone answered “yes” to any question in ASQ, they were considered as a “positive” screen and were evaluated by a psychologist or social worker in the clinic at the same visit. A brief suicide safety assessment (BSSA) was conducted to determine if a more comprehensive mental health evaluation was needed. Safety planning was performed using a written safety plan or a “Suicide Safety Plan” application, and support and resources were provided as indicated. The decision to use a written safety plan or the “Suicide Safety Plan” app was made by the psychologist and social worker, based on the patient's age and phone availability.

Participants who screened positive were required to complete the necessary steps before leaving the clinic (Figure 1).

**Figure 2:**
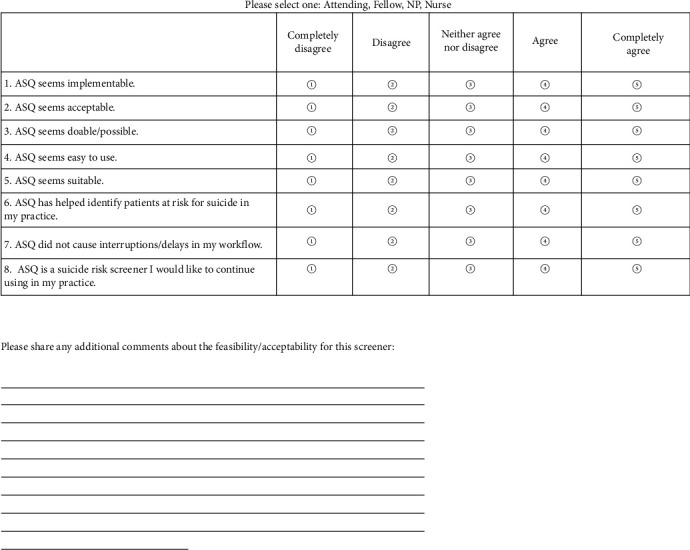
Suicide risk screening feasibility and acceptability survey.

To assess provider acceptability and feasibility of administering the new assessment tool (ASQ) in clinic, an anonymous survey was conducted at the conclusion of the study (Figure 2).

### 2.3. Data Collection

Study data were collected and managed using the Research Electronic Data Capture (REDCap) tool hosted at Johns Hopkins University [24]. Data collected include patient demographics (age, sex, race, ethnicity), clinical characteristics, and history of diabetes (type, duration, medications, technology use, hospitalizations), mental health history (from medical record and self-reported), mental health-related medications (specifically antidepressants), social determinants of heath (parental education, household income, insurance), and PHQ-9 and ASQ scores. Parental education and household income were obtained, where available, from another IRB-approved study.

### 2.4. Statistical Analysis

Patient demographic and clinical characteristics were compared in terms of diabetes type and in relation to positive or negative PHQ-9 and ASQ results using Fisher's exact tests and two sample *t*-tests. The diagnostic validity of the two questionnaires (PHQ-9 and ASQ) was assessed using the ASQ as the reference standard. Sensitivity, specificity, positive and negative predictive values, and false-positive and false-negative rates, and 95% confidence intervals were calculated. A post-hoc sensitivity analysis with and without ASQ Question 4 was conducted to understand the impact of this question on the validity analysis. ASQ Question 4 states, “Have you ever tried to kill yourself?” which assesses passive suicide risk.

A series of adjusted and unadjusted logistic regression models were generated to understand the correlation between suicide risk, depression, diabetes type, and demographic and clinical characteristics. Suicide risk was defined as positive if there was a positive ASQ and/or positive PHQ-9 Item 9. Adjusted models included diabetes type, age, gender, race, insurance, duration of diabetes, metformin use, GLP-1 agonist use, family history of mental health disorder, and personal history of mental health disorder (per the electronic health record). Follow-up with a mental health provider was assessed at 1-, 3-, and 6 months after the initial suicide risk screening. A cutoff of *p* < 0.05 was used to determine significance. All analyses were conducted using Stata software, version 18.0 [25].

## 3. Results

### 3.1. Patient Demographics

A total of 332 patients with T1D and T2D were given PHQ-9 and ASQ for this study. After excluding 23 patients with incomplete ASQ forms, the total number included in our study was 309. Of the 309 patients, 237 had T1D and 72 had T2D. As shown in Table 1, in patients with T1D, the mean age was 14.8 ± 2.5 years, 106 (44.7%) were female, 141 (59.5%) were White, 74 (31.2%) were Black, and 82 (34.9%) had public insurance with a mean HbA1c of 8.7 ± 2.2%. Among the 152 patients on insulin pumps, 131 (86.2%) were on a hybrid closed-loop pump. Among the subset of T1D patients (*n* = 59) with social determinants of health data, 11 (18.6%) had a household income of $49,999 or less, and 25 (42.4%) had parental education of high school or less.

The mean age for patients with T2D was 16.1 ± 2.8 years, 39 (54.2%) were female, 53 (73.6%) were Black, 11 (15.3%) were Hispanic/Latino and 60 (83.3%) had public insurance. The mean HbA1c was 8.4 ± 2.7%, 41 (56.9%) were on insulin, 61 (84.7%) on metformin, and 25 (34.7%) on a GLP-1 agonist. Among the subset of T2D patients with social determinants of health data (*n* = 23), 14 (60.8%) had a household income of $49,999 or less, and 14 (60.8%) had parental education of high school or less.

### 3.2. Baseline Mental Health Characteristics

In T1D patients, 60 (25.3%) had a mental health diagnosis recorded in the EMR with the majority having depression (*n* = 35,14.8%) and ADHD (*n* = 30,12.7%), 55 (23.2%) were regularly seen by a behavioral health provider, 20 (32.3%) had an antipsychotic/antidepressant prescription, and 92 (38.8%) had a family history of mental health disorder. Overall, 27.8% (66/237) of patients with T1D reported some degree of depression (PHQ-9> = 5), 5.9% (14/237) were positive for suicide risk on PHQ-9 Item 9, and 8.4% (20/237) were positive for suicide risk on ASQ (Table 2).

In T2D patients, 33 (45.8%) had a mental health diagnosis in the EMR with the majority having depression (*n* = 20, 27.8%) and ADHD (*n* = 17, 23.6%), 24 (33.3%) were regularly seen by a behavioral health provider, 14 (41.2%) had an antipsychotic/antidepressant prescription, and 35 (48.6%) had a family history of mental health disorder. Overall, 50% (36/72) of patients with T2D reported some degree of depression on the PHQ-9. For suicide risk screening, 12.5% (9/72) were positive for PHQ-9 Item 9, and 19.4% (14/72) were positive on the ASQ (Table 2). Further delineation of ASQ responses by each question is detailed in Table S1.

Patients with T2D had higher rates of mental health diagnosis (45.8% vs 25.3%, *p*=0.001), higher rates of depression on the PHQ-9 (50% vs 27.8%, *p*=0.001), and higher rates of ASQ positivity (19.4% vs 8.4%, *p*=0.016) compared to patients with T1D (Table S2).

### 3.3. Sensitivity and Specificity of the PHQ-9 Item 9 Compared to ASQ

As shown in Tables 3 and 4, the diagnostic accuracy of the PHQ-9 Item 9 was compared to the ASQ risk screener. In patients with T1D, as compared to the ASQ, the sensitivity of PHQ-9 Item 9 was 60% (95% CI: 36.1, 80.9%), specificity was 99.1% (95% CI: 96.7, 99.9%), PPV was 85.7% (95% CI: 57.2, 98.2%), and NPV was 96.4% (95% CI: 93.1, 98.4%).

As compared to the ASQ, the sensitivity of PHQ-9 Item 9 in patients with T2D was 50% (95% CI: 23, 77%), specificity was 96.6% (95% CI: 88.1, 99.6%), PPV was 77.8% (95% CI: 40, 97.2%), and NPV was 88.9% (95% CI: 78.4, 95.4%).

Of the 34 participants identified for suicide risk by ASQ (including Question 4), 19 screened positive for suicide risk on the PHQ-9 Item 9. The PHQ-9 Item 9 correctly identified the absence of suicide risk in 271 of 290 participants, who did not report suicide risk on the ASQ. The PHQ-9 Item 9 overidentified 1.5% (4/275) of patients at risk, who did not report suicide risk on the ASQ (Table 3).

ASQ Question 4 addresses any previous suicide attempt and thus always remains positive. Given this, we also performed a post-hoc exploratory analysis of sensitivity and specificity of the PHQ-9 Item 9 in comparison to the ASQ, excluding Question 4. In T1D youth, when ASQ question four was excluded, the sensitivity of PHQ-9 Item 9 was 91.7% (95% CI: 61.5, 99.8%), specificity was 98.7% (95% CI: 96.2, 99.7%), PPV was 78.6% (95% CI: 49.2, 95.3%), and NPV was 99.6% (95% CI: 97.5, 100%) as compared to ASQ. In T2D youth, when ASQ Question 4 was excluded, the sensitivity of PHQ-9 Item 9 was 85.7% (95% CI: 42.1, 99.6%), specificity was 95.4% (95% CI: 87.1, 99%), PPV was 66.7% (95% CI: 29.9, 92.5%), and NPV was 98.4% (95% CI: 91.5, 100%) as compared to ASQ (Table 4).

### 3.4. Univariate and Multivariate Analysis

As shown in Table S2, on univariate logistic analysis, being female, having a diagnosis of T2D, any depression, prior mental health disorders, regularly seen by behavioral health, and family history of mental health disorders were all significantly associated with suicide risk as measured by PHQ-9 Item 9 or ASQ (*p* < 0.01). On multivariate logistic analysis, after adjusting for age, race, gender, type of diabetes, insurance, and mental health disorders, individuals of female gender (OR 4.70, 95% CI: 1.76, 12.54, *p*=0.002), prior mental health disorders (OR 24.43, 95% CI: 8.12, 73.54, *p*=0.001), and family history of mental health disorder (OR 2.84, 95% CI: 1.03, 7.86, *p*=0.044) had greater odds of suicide risk (Table S3).

Factors associated with the outcome of depression (as measured by PHQ-9) are shown in Table S4. On univariate logistic analysis, being female, having a diagnosis of T2D, Black race, positive suicide screen on PHQ-9 Item 9 or ASQ, prior mental health disorders, regularly seen by behavioral health and family history of mental health disorders, were all significantly associated with outcome of depression (*p* < 0.01). Private insurance was inversely associated with the outcome of depression (OR 0.47, 95% CI: 0.29, 0.76, *p*=0.002).

After adjusting for age, race, gender, type of diabetes, insurance, mental health disorders in a multivariate logistic analysis, age (OR 0.87, 95% CI: 0.77, 0.99, *p*=0.04), black race (OR 2.45, 95% CI: 1.19,5.04, *p*=0.014), family history of mental health disorder (OR 2.48, 95% CI: 1.32,4.66, *p*=0.005), and prior mental health disorders (OR 5.59, 95% CI: 2.99, 10.42, *p*=0.001) had greater odds of depression (Table S5).

### 3.5. Follow-Up of Patients With Suicide Risk

Of the 34 people identified as positive suicide risks on ASQ, 33 were seen by a social worker and/or a psychologist/behavioral health provider in the clinic during their diabetes care visit. One individual who was positive on ASQ Question 4 (past suicide risk), was assessed, and did not have concerns for active SI or recent SI and thus was followed up by their regular outpatient mental health provider. One person was positive for all ASQ questions and was determined to be actively suicidal (ASQ Question 5 positive) and was sent to the emergency department to be evaluated. Among the 34 patients who screened positive on ASQ, 64.7% were regularly seen by behavioral health prior to this visit with 46.4% on an antipsychotic/antidepressant medication, and 64.7% had a family history of mental health disorder (depression, mood disorder, or anxiety).

Among the 34 who were identified as ASQ positive, 18/34 (52.9%) followed up with an outpatient mental health provider within 1 month, 21/34 (61.7%) followed up within 3 months, and 12/34 (35.3%) followed up within 6 months.

### 3.6. Provider Feasibility and Acceptability Survey

**Figure 3:**
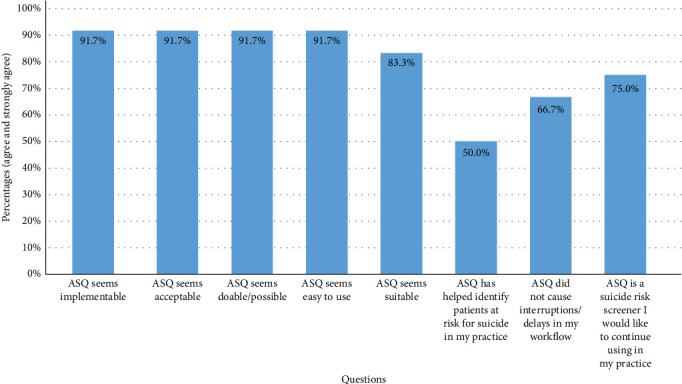
Feasibility and acceptability survey results.

Providers in the diabetes clinic were surveyed to assess the feasibility and acceptability of using ASQ in routine clinic visits. Of the 12 participants that responded, eight were providers (seven endocrinologists, one nurse practitioner), and four were pediatric endocrine fellows. As shown in Figure 3, most providers reported that ASQ was easy to implement and use, and 75% of the providers would continue to use ASQ in their practice. Of the respondents, 66.7% stated that ASQ did not cause delays in their workflow, and 50% stated that ASQ helped identify patients at risk for suicide.

## 4. Discussion

To our knowledge, this is the first study comparing the diagnostic accuracy of PHQ-9 Item 9 to the ASQ as a reference standard for suicide risk screening in an ethnically and racially diverse group of AYA with T1D and T2D. In T1D youth, the sensitivity and specificity of PHQ-9 Item 9 compared with ASQ were 60% and 99.1%, respectively. In T2D youth, the sensitivity and specificity of PHQ-9 Item 9 compared with ASQ were 50% and 96.6%, respectively. Thus, reliance on PHQ-9 Item 9 is not sufficient to identify suicide risk, and use of a suicide risk screener is preferred to accurately identify at-risk youth. Further, the prevalence of depression and suicide was higher than expected in this youth population, particularly in the T2D cohort, highlighting the importance of screening for mental health disorders at routine diabetes clinic visits. A significant concern in youth with T1D is the relatively easy access to potentially lethal means of suicide, specifically insulin, which has been reported as a method of suicide attempt in this population [26].

In our study, we found higher rates of depression (PHQ-9 score ≥ 5) in youth with T2D compared with T1D (50% vs 27.8%), similar to the PDC study that also reported a 50% higher depression rate in adolescents with T2D compared with T1D [6]. Several studies, including the Treatment Options for T2D in Adolescent and Youth (TODAY) study, have shown depression rates of 17%–31% in AYA with T2D [11, 13, 27], although we saw higher rates in this T2D cohort at 50%. The higher depression rates we observed may be attributed to the aftermath of the COVID-19 pandemic, or the general rise in mental health challenges, or the sampling from a racially diverse and socioeconomically disadvantaged population [28]. Adolescents from socioeconomically disadvantaged backgrounds, particularly those living in high-crime neighborhoods with limited access to recreational facilities, face an increased likelihood of experiencing elevated stress and depressive symptoms. Additionally, systemic challenges, including racial and ethnic discrimination and social marginalization, significantly contribute to the worsening of mental health issues among this population [29, 30].

Several research studies have employed specific suicide risk screening tools in routine diabetes clinics to assess the rates of suicide risk, namely ASQ, C-SSRS, and SIQ/SIQ-JR while others have used the PHQ-9 Item 9. In AYA with T1D the observed rates of suicide risk reported in the literature are 2.2%–15.0% [9, 10]. We observed lower rates of suicide risk in T1D, with 5.9% on PHQ-9 Item 9, and 8.4% on ASQ. A recent study published in 2022 assessing suicide risk comparing PHQ-9 Item 9 with C-SSRS as the reference standard found a suicide risk of 11.3% in AYA with T1D on the C-SSRS [9]. In a Canadian population study in 2008, the prevalence of SI among those with T1D was 15.0% compared with 9.4% in patients without T1D [10]. We may have observed lower suicide risk rates in this cohort because a high proportion of our population (64%) were using diabetes technology, which is shown to be associated with improved quality of life and lower diabetes burden [31]. Among T1D, 86.2% were on hybrid closed loop insulin pumps, and according to a study by Cobry et al. [31] published in 2021, children on hybrid closed loop systems did not experience increased diabetes burden.

In this cohort of AYA with T2D, the observed rate of suicide risk was 12.5% using PHQ-9 Item 9, and 19.4% using ASQ, similar to rates reported in the literature of 9%–18.9% [11–13]. In a study performed at 4 academic centers, among a subset of 53 individuals with T2D, 18.9% endorsed thoughts of self-harm [13]. While our rates of suicide risk using ASQ were similar to this study, our population was larger and more diverse (73.6% Black), compared to this other study (49.2% Black). Additionally, children with T2D were more likely to have a mental health disorder before and after diagnosis, to attempt suicide and be prescribed an antipsychotic compared to youth with T1D [12], which was similar to the trends we saw in this population.

Given the high rates of depression and suicide risk, our goal was to determine the diagnostic accuracy of PHQ-9 Item 9 compared to ASQ as the gold standard. Interestingly, the PHQ-9 Item 9 sensitivity was low (55.9%) compared to the ASQ. We believe this is due to ASQ inclusion of ASQ Question 4 which asks about prior suicide attempts and is also considered a risk for suicide. Thus, any individual with history of a previous attempt will be identified on ASQ but would be missed with sole use of PHQ-9 Item 9. Therefore, we also analyzed the diagnostic accuracy without ASQ Question 4 and found that the sensitivity of PHQ-9 Item 9 was 89.5%. As we hypothesized, the ASQ had improved sensitivity to detect suicide risk when compared to PHQ-9 Item 9, and this is likely due to the inclusion of ASQ Question 4 which asks about prior suicide attempt, which is a strong risk factor for suicide risk. This is similar to other studies comparing PHQ-9 Item 9 to other suicide-risk screeners, including a comparison to the C-SSRS in T1D adolescents, which showed a PHQ-9 Item 9 sensitivity of 53.3% [9], and a comparison to the SIQ/SIQ-JR, where the PHQ-9 Item 9 had a sensitivity of 70% in pediatric inpatients [32]. The C-SSRS and SIQ/SIQ-JR are longer questionnaires (19 and 15 items, respectively) and although we did not directly compare ASQ with C-SSRS or the SIQ/SIQ-JR, given the brevity of ASQ and the similar sensitivity to other suicide risk screeners compared with the PHQ-9 Item 9, we would continue the use of ASQ for detecting suicide risk. Furthermore, most healthcare providers in our clinic found it feasible to implement and identify high-risk patients without having negative impacts on workflow.

Consistent with previous research, we demonstrated higher odds of suicide risk in females compared to males. A CDC report in 2023, assessing emergency department visits with SI found that females aged 14–18 years had the highest rates of suicidality [33]. In a review article discussing disparities in pediatric mental health conditions, increased rates of suicide were observed in black adolescent females and younger males [34]. This further highlights the importance of suicide risk screening, particularly in clinic settings with a racially and ethnically diverse population.

In this study, among the patients identified as at risk for suicide, the majority had seen a behavioral health specialist prior to the visit and almost half of the patients were on psychotropic medications. These numbers are higher than those reported by Majidi et al. [35] but this may be due to the inclusion of both T1D and T2D in this study. Acute intervention at the time of a positive suicide risk screening is important to ensure the safety of patients completing these questionnaires. This clinic is fortunate to have a social worker and behavioral health psychology team available to address these concerns at the point of care. Although all patients were referred for mental health follow-up, very few completed this follow-up within 1 month and not all completed follow-up within 6 months. A notable observation is that, despite higher reported suicide risk, fewer youth with T2D sought mental health services at all three follow-up time points reviewed. This highlights the importance of having behavioral health teams embedded in multidisciplinary diabetes clinics to address psychosocial concerns, especially suicide risk, as it is challenging to access outpatient mental health resources.

A strength of this study is the evaluation of suicide risk in AYA with diabetes in an ethnically and racially diverse population, making the results more generalizable. Additionally, ASQ is a four-question tool, and takes less than 1 min to complete, making it easy, and feasible to administer.

There are several limitations of this study. Firstly, it is a single site study, performed at a tertiary care hospital, where our diverse population may not be similar to other clinic settings. Although we used insurance data (Public v Private) as a proxy for social determinants of health, data on household income and parental education were only available in a subset of the patients, which limited the ability to further analyze these SDOH risk factors. Furthermore, almost half of the patients with positive ASQ screeners had ASQ Question 4 positive, which is an assessment of a past suicide attempt and is considered an enduring suicide risk factor but may not reflect current and active suicide risk.

## 5. Conclusion

Diabetes is a chronic disease with high rates of depression and suicide risk. Our results illustrate the need to assess depression and suicide risk for individuals with diabetes. Given the higher sensitivity and brevity of the ASQ, it is an efficient and feasible screener to utilize in routine diabetes care, especially compared to the lower sensitivity of the PHQ-9 Item 9. Future studies should investigate means to improve follow-up with mental health services and evaluate depression and suicide risk in larger cohorts and evaluate methods of intervention and follow-up.

## Figures and Tables

**Table 1:** Demographics and clinical diabetes characteristics.

Variable	All	T1D	T2D	*p*-value
*N*	309	237	72	—
Age, mean (SD)	15.1 (2.6)	14.8 (2.5)	16.1 (2.8)	<0.001
Gender	—	—	—	0.058
Male	161 (52.1%)	130 (54.9%)	31 (43.1%)	—
Female	145 (46.9%)	106 (44.7%)	39 (54.2%)	—
Other	3 (1.0%)	1 (0.4%)	2 (2.8%)	—
Race	—	—	—	<0.001
White	146 (47.2%)	141 (59.5%)	5 (6.9%)	—
Black	127 (41.1%)	74 (31.2%)	53 (73.6%)	—
Asian	5 (1.6%)	5 (2.1%)	0 (0.0%)	—
Other	25 (8.1%)	14 (5.9%)	11 (15.3%)	—
Unknown	6 (1.9%)	3 (1.3%)	3 (4.2%)	—
Ethnicity	—	—	—	<0.001
Non-Hispanic/Latino	278 (90.0%)	222 (93.7%)	56 (77.8%)	—
Hispanic/Latino	18 (5.8%)	7 (3.0%)	11 (15.3%)	—
Unknown	13 (4.2%)	8 (3.4%)	5 (6.9%)	—
Insurance status	—	—	—	<0.001
Public	142 (46.3%)	82 (34.9%)	60 (83.3%)	—
Private	164 (53.4%)	152 (64.7%)	12 (16.7%)	—
Household income (*n* = 82)	—	—	—	0.003
$25,000 or less	14 (17.1%)	7 (11.9%)	7 (30.4%)	—
$25,000–$49,999	11 (13.4%)	4 (6.8%)	7 (30.4%)	—
$50,000 or more	39(47.6%)	35(59.3%)	4 (17.4%)	—
Unknown	18 (22.0%)	13 (22.0%)	5 (21.7%)	—
Parental Education (*n* = 82)	—	—	—	0.054
High school or less	39 (47.6%)	25 (42.4%)	14 (60.9%)	—
Beyond high school	34 (41.5%)	29 (49.2%)	5 (21.7%)	—
Unknown	9 (11.0%)	5 (8.5%)	4 (17.4%)	—
Diabetes duration, mean (SD) (*n* = 309)	4.3 (3.7)	4.9 (3.9)	2.3 (1.4)	<0.001
DKA at diagnosis	140 (47.5%)	126 (56.0%)	14 (20.0%)	<0.001
HbA1c at diagnosis, mean (SD) (*n* = 292)	11.7 (2.6)	11.9 (2.4)	11.2 (3.2)	0.048
Current HbA1c, mean (SD) (*n* = 309)	8.6 (2.3)	8.7 (2.2)	8.4 (2.7)	0.35
Insulin delivery method	—	—	—	<0.001
Not taking insulin	31 (10.0%)	0 (0.0%)	31 (43.1%)	—
Insulin pump	152 (49.2%)	152 (64.1%)	0 (0.0%)	—
Hybrid closed-loop insulin pump	131 (86.2%)	131 (86.2%)	N/A	—
Injections	126 (40.8%)	85 (35.9%)	41 (56.9%)	—
CGM use	220 (71.2%)	208 (87.8%)	12 (16.7%)	<0.001
Metformin	61 (19.7%)	N/A	61 (84.7%)	<0.001
GLP-1 agonist	25 (8.1%)	N/A	25 (34.7%)	<0.001

*Note:* Household income and parental education data were only available for a subset of patients (*n* = 82).

Abbreviations: CGM, continuous glucose monitor; DKA, diabetic ketoacidosis; HbA1c, hemoglobin A1c.

**Table 2:** Mental health characteristics and screening results.

Variable	All	T1D	T2D	*p*-Value
PHQ-9 positive for depression (PHQ-9 score ≥ 5)	102 (33.0%)	66 (27.8%)	36 (50.0%)	<0.001
PHQ-9 score category	—	—	—	<0.001
Mild (5–9)	60 (19.4%)	44 (18.6%)	16 (22.2%)	—
Moderate (10–14)	24 (7.8%)	13 (5.5%)	11 (15.3%)	—
Moderately severe (15–19)	14 (4.5%)	6 (2.5%)	8 (11.1%)	—
Severe (20–27)	4 (1.3%)	3 (1.3%)	1 (1.4%)	—
PHQ-9 Item 9 positive	23 (7.4%)	14 (5.9%)	9 (12.5%)	0.062
ASQ positive	34 (11.0%)	20 (8.4%)	14 (19.4%)	0.009
Mental health diagnosis in EMR	93 (30.1%)	60 (25.3%)	33 (45.8%)	<0.001
Regularly seen by behavioral health (yes)	79 (25.6%)	55 (23.2%)	24 (33.3%)	0.085
Family history of mental health disorders	—	—	—	0.33
Yes	127(41.1%)	92 (38.8%)	35 (48.6%)	—
Unknown	40 (12.9%)	32 (13.5%)	8 (11.1%)	—
Antipsychotic medication prescription	34 (35.4%)	20 (32.3%)	14 (41.2%)	0.38

Abreviation: EMR, electronic medical record.

**Table 3:** Sensitivity and specificity of PHQ-9 Item 9 as compared to the ASQ (*n* = 309).

Patients	Sensitivity	Specificity	PPV	NPV
All patients with diabetes	55.9% (CI 37.9%,72.8%)	98.5% (CI 96.3%,99.6%)	82.6% (CI 61.2%, 95%)	94.8% (CI 91.5%, 97%)
Type 1 diabetes	60% (CI 36.1%, 80.9%)	99.1% (CI 96.7%, 99.9%)	85.7% (CI 57.2%, 98.2%)	96.4% (CI 93.1%, 98.4%)
Type 2 diabetes	50% (CI 23%, 77%)	96.6% (CI 88.1%, 99.6%)	77.8% (CI 40%, 97.2%)	88.9% (CI 78.4%, 95.4%)

Abbreviations: NPV, negative predictive value; PPV, positive predictive value.

**Table 4:** Sensitivity and specificity of PHQ-9 Item 9 as compared to ASQ excluding question 4.

Patients	Sensitivity	Specificity	PPV	NPV
Excluding ASQ question 4				
All patients with diabetes	89.5% (CI 66.9%,98.7%)	97.9% (CI 95.6%, 99.2%)	73.9% (CI 51.6%, 89.8%)	99.3% (CI 97.5%, 99.9%)
Type 1 diabetes	91.7% (CI 61.5%,99.8%)	98.7% (CI 96.2%, 99.7%)	78.6% (CI 49.2%, 95.3%)	99.6% (CI 97.5%, 100%)
Type 2 diabetes	85.7% (CI 42.1%,99.6%)	95.4% (CI 87.1%, 99%)	66.7% (CI 29.9%, 92.5%)	98.4% (CI 91.5%, 100%)

Abbreviations: NPV, negative predictive value; PPV, positive predictive value.

## Data Availability

The data that support the findings of this study are available from the corresponding author upon reasonable request. Due to the sensitive nature of the data, access is restricted and will require a formal written application. Requests will be subject to institutional approvals and compliance with ethical and data protection regulations.

## Supporting Information

Supporting Information Table S1. Detailed scoring of PHQ-9 and ASQ and mental health follow-up data—2. Table S2. Univariate logistic analysis with outcome of positive suicide risk—3,4. Table S3. Multivariate logistic analysis with positive suicide risk as outcome—5. Table S4. Univariate logistic analysis with outcome of depression —6,7. Table S5. Multivariate logistic analysis with depression as outcome —8.
