# Supplementary material for: Suicide Risk Screening in a Diverse Cohort of Youth With Type 1 and Type 2 Diabetes
Source: Pediatr Diabetes. 2025 Jun 2;2025:6662248. doi: 10.1155/pedi/6662248 (PMC12149509; doi:10.1155/pedi/6662248)
Supplement: Supporting Information — Table S1. Detailed scoring of PHQ-9 and ASQ and mental health follow-up data—2. Table S2. Univariate logistic analysis with outcome of positive suicide risk—3,4. Table S3. Multivariate logistic analysis with positive suicide risk as outcome—5. Table S4. Univariate logistic analysis with outcome of depression —6,7. Table S5. Multivariate logistic analysis with depression as outcome —8. [file 6662248.f1.docx]

**Suicide Risk Screening and Poor Follow-up in Diverse Youth with Type 1 and 2 Diabetes**

**(Supplement)**

Table S1: Detailed Scoring of PHQ-9 and ASQ and mental health follow up data………………..2
Table S2:Univariate logistic analysis with outcome of positive suicide risk……………………...3,4
Table S3: Multivariate logistic analysis with positive suicide risk as outcome………………....…..5
Table S4: Univariate logistic analysis with outcome of depression ……………………………....6,7
Table S5: Multivariate logistic analysis with depression as outcome ………………………………8

**Table S1: Detailed Scoring of PHQ-9 and ASQ and mental health follow up data.**

| **Factor** | **All** | **Type 1** | **Type 2** | **p-value** |
| --- | --- | --- | --- | --- |
| N | 309 | 237 | 72 |  |
| PHQ-9 Score Category |  |  |  | <0.001 |
| None | 207 (67.0%) | 171 (72.2%) | 36 (50.0%) |  |
| Mild | 60 (19.4%) | 44 (18.6%) | 16 (22.2%) |  |
| Moderate | 24 (7.8%) | 13 (5.5%) | 11 (15.3%) |  |
| Moderately severe | 14 (4.5%) | 6 (2.5%) | 8 (11.1%) |  |
| Severe | 4 (1.3%) | 3 (1.3%) | 1 (1.4%) |  |
| PHQ-9 Positive for depression | 102 (33.0%) | 66 (27.8%) | 36 (50.0%) | <0.001 |
| PHQ-9 Item 9 | 23 (7.4%) | 14 (5.9%) | 9 (12.5%) | 0.074 |
| ASQ question (1) =Yes | 10 (3.2%) | 7 (3.0%) | 3 (4.2%) | 0.70 |
| ASQ question (2) =Yes | 16 (5.2%) | 10 (4.2%) | 6 (8.3%) | 0.22 |
| ASQ question (3) =Yes | 8 (2.6%) | 4 (1.7%) | 4 (5.6%) | 0.089 |
| ASQ question (4) =Yes | 21 (6.8%) | 13 (5.5%) | 8 (11.1%) | 0.11 |
| ASQ question (5) =Yes | 1 (2.9%) | 1 (5.0%) | 0 (0.0%) | 1.00 |
| ASQ positive | 34 (11.0%) | 20 (8.4%) | 14 (19.4%) | 0.016 |
| ASQ 1 question positive | 23 (7.4%) | 12 (5.1%) | 11 (15.3%) | 0.008 |
| ASQ 2 questions positive | 5 (1.6%) | 5 (2.1%) | 0 (0.0%) | 0.59 |
| ASQ 3 questions positive | 2 (0.6%) | 0 (0.0%) | 2 (2.8%) | 0.054 |
| ASQ 4 questions positive | 3 (1.0%) | 2 (0.8%) | 1 (1.4%) | 0.55 |
| ASQ 5 questions positive | 1 (0.3%) | 1 (0.4%) | 0 (0.0%) | 1.00 |
| ASQ only question 4 positive | 15 (4.9%) | 8 (3.4%) | 7 (9.7%) | 0.053 |
| Other ASQ question positive (not 4) | 19 (6.1%) | 12 (5.1%) | 7 (9.7%) | 0.16 |
| Positive for either test | 38 (12.3%) | 22 (9.3%) | 16 (22.2%) | 0.007 |
| Followed by mental health within 1 month | 18 (52.9%) | 13 (72.2%) | 5 (38.5%) |  |
| Followed by mental health within 3 months | 21 (61.7%) | 14 (66.7%) | 7 (33.3%) |  |
| Followed by mental health within 6 months | 12 (35.3%) | 8 (66.7%) | 4 (33.3%) |  |

**Table S2:** **Univariate logistic analysis with outcome of positive suicide risk.**

| **Variable** | **Odds Ratio** | **[95% Conf.** | **Interval]** | **P>\|z\|** |
| --- | --- | --- | --- | --- |
|  |  |  |  |  |
| Diabetes Type |  |  |  |  |
| Type 1 | Reference |  |  |  |
| Type 2 | 2.792 | 1.376 | 5.668 | **0.004** |
| Age | 0.984 | 0.864 | 1.120 | 0.803 |
| Gender |  |  |  |  |
| Male | Reference |  |  |  |
| Female | 5.739 | 2.435 | 13.528 | **0.000** |
| Race |  |  |  |  |
| White | Reference |  |  |  |
| Black | 2.027 | 0.970 | 4.237 | 0.060 |
| Asian | 1.000 | -- |  | - |
| Other | 0.890 | 0.188 | 4.205 | 0.883 |
| Unknown/Not reported | 5.115 | 0.854 | 30.650 | 0.074 |
| Ethnicity |  |  |  |  |
| Non-Hispanic/Latino | Reference |  |  |  |
| Hispanic/Latino | 1.485 | 0.408 | 5.404 | 0.549 |
| Unknown/Not Reported | 1.350 | 0.287 | 6.359 | 0.704 |
| Insurance |  |  |  |  |
| Public | Reference |  |  |  |
| Private | 0.666 | 0.336 | 1.320 | 0.244 |
| None/Unknown | 1.000 | (empty) |  |  |
| Income category |  |  |  |  |
| Less than $50,000 | Reference |  |  |  |
| $50,000-$99,999 | 0.257 | 0.047 | 1.401 | 0.116 |
| $100,000 or more | 0.343 | 0.062 | 1.904 | 0.221 |
| No response | 0.321 | 0.058 | 1.777 | 0.193 |
| Highest parental education |  |  |  |  |
| High school or less | Reference |  |  |  |
| More than high school | 0.442 | 0.105 | 1.867 | 0.267 |
| No response | 2.286 | 0.457 | 11.426 | 0.314 |
| BMI | 1.046 | 1.010 | 1.083 | **0.012** |
| BMI Percentile | 1.014 | 0.999 | 1.029 | 0.071 |
| Age at diagnosis | 1.034 | 0.949 | 1.126 | 0.448 |
| Diabetes duration | 0.948 | 0.856 | 1.051 | 0.311 |
| DKA at diagnosis | 0.725 | 0.360 | 1.461 | 0.369 |
| HbA1c at diagnosis | 1.024 | 0.892 | 1.174 | 0.741 |
| Current HbA1c | 1.067 | 0.927 | 1.228 | 0.365 |
| Insulin delivery Method |  |  |  |  |
| Not taking insulin | Reference |  |  |  |
| Insulin Pump | 0.685 | 0.209 | 2.240 | 0.531 |
| Injections | 1.274 | 0.402 | 4.037 | 0.681 |
| Metformin | 2.780 | 1.339 | 5.771 | **0.006** |
| GLP-1 agonist | 3.174 | 1.228 | 8.201 | **0.017** |
| PHQ-9 Score Category |  |  |  |  |
| None | Reference |  |  |  |
| Mild | 7.520 | 2.651 | 21.332 | **0.000** |
| Moderate | 23.929 | 7.592 | 75.420 | **0.000** |
| Moderately severe | 33.500 | 8.900 | 126.095 | **0.000** |
| Severe | 1.000 | (empty) |  |  |
| PHQ-9 Positive for depression | 15.314 | 6.144 | 38.175 | **0.000** |
| Mental health diagnosis in EMR | 18.361 | 7.336 | 45.951 | **0.000** |
| Depression (per EMR) | 36.900 | 15.279 | 89.119 | **0.000** |
| Anxiety (per EMR) | 4.482 | 1.909 | 10.525 | **0.001** |
| ADHD (per EMR) | 3.625 | 1.694 | 7.754 | **0.001** |
| Mood disorder (per EMR) | 3.786 | 0.906 | 15.820 | 0.068 |
| Regularly seen by Behavioral health |  |  |  |  |
| No | Reference |  |  |  |
| Yes | 8.912 | 4.222 | 18.810 | **0.000** |
| Family History of mental health disorders |  |  |  |  |
| No | Reference |  |  |  |
| Yes | 4.105 | 1.778 | 9.479 | **0.001** |
| Unknown | 2.393 | 0.737 | 7.769 | 0.146 |
| Antipsychotic medication/prescription | 3.133 | 1.288 | 7.620 | **0.012** |
| Hospitalizations for Mental Health Related Issues |  |  |  |  |
| 0 visit | Reference |  |  |  |
| 1-2 visits | 2.286 | 0.778 | 6.714 | 0.133 |
| >=3 visits | 1.000 | (empty) |  |  |

HbA1c: hemoglobin A1c, DKA: diabetic ketoacidosis, BMI: body mass index, GLP-1: glucagon-like peptide 1, EMR: electronic medical record.

**Table S3: Multivariate logistic analysis with positive suicide risk as outcome.**

| **Variable** | **Odds Ratio** | **[95% Conf.** | **Interval]** | **P>\|z\|** |
| --- | --- | --- | --- | --- |
|  |  |  |  |  |
| Diabetes Type |  |  |  |  |
| Type 1 | Reference |  |  |  |
| Type 2 | 1.677 | 0.180 | 15.592 | 0.650 |
| Age | 0.915 | 0.747 | 1.120 | 0.388 |
| Gender |  |  |  |  |
| Male | Reference |  |  |  |
| Female | 4.700 | 1.761 | 12.548 | 0.002 |
| Other | 8.836 | 0.129 | 604.117 | 0.312 |
| Race |  |  |  |  |
| White | Reference |  |  |  |
| Black | 2.201 | 0.666 | 7.267 | 0.196 |
| Other/Unknown | 2.685 | 0.480 | 15.010 | 0.261 |
| Insurance |  |  |  |  |
| Public | Reference |  |  |  |
| Private | 2.313 | 0.770 | 6.950 | 0.135 |
| None/Unknown | 1.000 |  |  |  |
| BMI | 1.018 | 0.953 | 1.088 | 0.593 |
| Diabetes duration | 0.920 | 0.791 | 1.069 | 0.275 |
| Metformin | 0.757 | 0.109 | 5.256 | 0.778 |
| GLP-1 agonist | 0.557 | 0.116 | 2.667 | 0.464 |
| Family history of mental health disorder |  |  |  |  |
| No | Reference |  |  |  |
| Yes | 2.848 | 1.031 | 7.866 | 0.044 |
| Unknown | 3.641 | 0.869 | 15.252 | 0.077 |
| Mental health diagnosis per EMR |  |  |  |  |
| No | Reference |  |  |  |
| Yes | 24.437 | 8.120 | 73.542 | 0.000 |

BMI: body mass index, GLP-1: glucagon-like peptide 1, EMR: electronic medical record.

**Table S4: Univariate logistic analysis with outcome of depression.**

| **Variable** | **Odds Ratio** | **[95% Conf.** | **Interval]** | **P>\|z\|** |
| --- | --- | --- | --- | --- |
|  |  |  |  |  |
| Diabetes Type |  |  |  |  |
| Type 1 | Reference |  |  |  |
| Type 2 | 2.591 | 1.506 | 4.456 | **0.001** |
| Age | 1.027 | 0.968 | 1.089 | 0.384 |
| Gender |  |  |  |  |
| Male | Reference |  |  |  |
| Female | 2.260 | 1.390 | 3.673 | **0.001** |
| Other | 1.000 |  |  |  |
| Race |  |  |  |  |
| White | Reference |  |  |  |
| Black | 2.666 | 1.591 | 4.466 | **0.000** |
| Asian | 4.757 | 0.764 | 29.629 | 0.095 |
| Other | 0.604 | 0.194 | 1.879 | 0.384 |
| Unknown/Not reported | 1.586 | 0.278 | 9.029 | 0.603 |
| Ethnicity |  |  |  |  |
| Non-Hispanic/Latino | Reference |  |  |  |
| Hispanic/Latino | 0.753 | 0.261 | 2.175 | 0.600 |
| Unknown/Not Reported | 0.587 | 0.158 | 2.185 | 0.427 |
| Insurance |  |  |  |  |
| Public | Reference |  |  |  |
| Private | 0.470 | 0.290 | 0.763 | **0.002** |
| None/Unknown | 1.000 |  |  |  |
| Income category |  |  |  |  |
| Less than $50,000 | Reference |  |  |  |
| $50,000-$99,999 | 0.830 | 0.247 | 2.791 | 0.763 |
| $100,000 or more | 0.547 | 0.137 | 2.189 | 0.394 |
| No response | 1.778 | 0.518 | 6.101 | 0.360 |
| Highest parental education |  |  |  |  |
| High school or less | Reference |  |  |  |
| More than high school | 0.492 | 0.177 | 1.368 | 0.174 |
| No response | 3.200 | 0.694 | 14.759 | 0.136 |
| BMI | 1.039 | 1.011 | 1.068 | **0.006** |
| BMI Percentile | 1.012 | 1.002 | 1.021 | **0.017** |
| Age at diagnosis | 1.027 | 0.968 | 1.089 | 0.384 |
| Diabetes duration | 0.938 | 0.875 | 1.006 | 0.074 |
| DKA at diagnosis | 0.784 | 0.482 | 1.274 | 0.326 |
| HbA1c at diagnosis | 1.002 | 0.913 | 1.100 | 0.963 |
| Current HbA1c | 1.143 | 1.033 | 1.266 | **0.010** |
| Insulin delivery Method |  |  |  |  |
| Not taking insulin | Reference |  |  |  |
| Insulin Pump | 0.495 | 0.222 | 1.100 | 0.084 |
| Injections | 0.881 | 0.397 | 1.958 | 0.756 |
| Metformin | 2.366 | 1.335 | 4.191 | **0.003** |
| GLP-1 agonist | 2.374 | 1.042 | 5.409 | **0.040** |
| Positive suicide screen ASQ/PHQ9-q9 | 15.314 | 6.144 | 38.175 | **0.000** |
| Mental health diagnosis in EMR | 5.204 | 3.079 | 8.796 | **0.000** |
| Depression (per EMR) | 5.977 | 3.184 | 11.220 | **0.000** |
| Anxiety (per EMR) | 4.079 | 1.859 | 8.949 | **0.000** |
| ADHD (per EMR) | 3.744 | 1.971 | 7.112 | **0.000** |
| Mood disorder (per EMR) | 2.616 | 0.687 | 9.959 | 0.159 |
| Regularly seen by Behavioral health |  |  |  |  |
| No | Reference |  |  |  |
| Yes | 4.331 | 2.526 | 7.425 | **0.000** |
| Family History of mental health disorders |  |  |  |  |
| No | Reference |  |  |  |
| Yes | 3.211 | 1.868 | 5.521 | **0.000** |
| Unknown | 3.331 | 1.577 | 7.035 | **0.002** |
| Antipsychotic medication/prescription | 0.915 | 0.393 | 2.128 | 0.836 |
| Hospitalizations for Mental Health Related Issues |  |  |  |  |
| 0 visit | Reference |  |  |  |
| 1-2 visits | 0.467 | 0.160 | 1.361 | 0.163 |
| >=3 visits | 1.000 | (empty) |  |  |

HbA1c: hemoglobin A1c, DKA: diabetic ketoacidosis, BMI: body mass index, GLP-1: glucagon-like peptide 1, EMR: electronic medical record

**Table S5: Multivariate logistic analysis with depression as outcome.**

| **Variable** | **Odds Ratio** | **[95% Conf.** | **Interval]** | **P>\|z\|** |
| --- | --- | --- | --- | --- |
|  |  |  |  |  |
| Diabetes Type |  |  |  |  |
| Type 1 | Reference |  |  |  |
| Type 2 | 2.073 | 0.400 | 10.742 | 0.385 |
| Age | 0.875 | 0.770 | 0.994 | 0.040 |
| Gender |  |  |  |  |
| Male | Reference |  |  |  |
| Female | 1.583 | 0.886 | 2.827 | 0.121 |
| Other | 1.000 |  |  |  |
| Race |  |  |  |  |
| White | Reference |  |  |  |
| Black | 2.459 | 1.199 | 5.045 | 0.014 |
| Other/Unknown | 1.571 | 0.540 | 4.570 | 0.407 |
| Insurance |  |  |  |  |
| Public | Reference |  |  |  |
| Private | 0.932 | 0.481 | 1.808 | 0.836 |
| None/Unknown | 1.000 |  |  |  |
| BMI | 1.012 | 0.963 | 1.064 | 0.625 |
| Diabetes duration | 0.940 | 0.857 | 1.031 | 0.190 |
| Metformin | 0.565 | 0.125 | 2.555 | 0.459 |
| GLP-1 agonist | 0.750 | 0.220 | 2.560 | 0.646 |
| Family history of mental health disorder |  |  |  |  |
| No | Reference |  |  |  |
| Yes | 2.485 | 1.325 | 4.663 | 0.005 |
| Unknown | 4.042 | 1.743 | 9.377 | 0.001 |
| Mental health diagnosis per EMR |  |  |  |  |
| No | Reference |  |  |  |
| Yes | 5.590 | 2.996 | 10.427 | 0.000 |
